# Assessing the Reliability and Validity of Principles for Health-Related Information on Social Media (PRHISM) for Evaluating Breast Cancer Treatment Videos on YouTube: Instrument Validation Study

**DOI:** 10.2196/66416

**Published:** 2025-06-11

**Authors:** Hiroki Kusama, Yoshimitsu Takahashi, Shunichiro Orihara, Kayo Adachi, Yumiko Ishizuka, Ryoko Semba, Hidetaka Shima, Yoshiya Horimoto, Hiroshi Kaise, Masataka Taguri, Sho Inoue, Takeo Nakayama, Takashi Ishikawa

**Affiliations:** 1 Department of Breast Surgical Oncology, Tokyo Medical University Hospital, 6-7-1, Nishishinjuku, Shinjuku-ku, Tokyo, 160-0023, Japan, 81 3-3342-6111; 2 Department of Implementation Science in Public Health, School of Public Health, Kyoto University, Kyoto, Japan; 3 Department of Health Data Science, Tokyo Medical University Hospital, Tokyo, Japan; 4 Department of Breast Oncology, Faculty of Medicine, Juntendo University, Tokyo, Japan; 5 Department of Breast Surgery, Douaikai Ozawa Hospital, Kanagawa, Japan; 6 Department of Breast Surgical Oncology, Tokyo Medical University Ibaraki Medical Center, Ibaraki, Japan; 7 YCU Co-Creation Innovation Center, Yokohama City University, Kanagawa, Japan; 8 Department of Health Informatics, School of Public Health, Kyoto University, Kyoto, Japan

**Keywords:** information quality, social media, YouTube, PRHISM, breast cancer treatment, videos, reliability, validity, instrument validation study, medical information, online health information, cancer treatment, Japan, Principles for Health-Related Information on Social Media

## Abstract

**Background:**

There is breast cancer–related medical information on social media, but there is no established method for objectively evaluating the quality of this information. Principles for Health-Related Information on Social Media (PRHISM) is a newly developed tool for objectively assessing the quality of health-related information on social media; however, there have been no reports evaluating its reliability and validity.

**Objective:**

The purpose of this study was to statistically examine the reliability and validity of PRHISM using videos about breast cancer treatment on YouTube (Google).

**Methods:**

In total, 60 YouTube videos were selected on January 5, 2024, with the Japanese words for “breast cancer,” “treatment,” and “chemotherapy,” and assessed by 6 Japanese physicians with expertise in breast cancer. These evaluators independently evaluated the videos using PRHISM and an established tool for assessing the quality of health-related information, DISCERN, as well as through subjective assessments. We calculated interrater and intrarater agreement among evaluators with CIs, measuring agreement using weighted Cohen kappa.

**Results:**

The interrater agreement for PRHISM overall quality was κ=0.52 (90% CI 0.49-0.55), indicating that the expected level of agreement, statistically defined by the lower limit of the 90% CI exceeding 0.53, was not achieved. However, PRHISM demonstrated higher agreement compared with DISCERN overall quality, which had a κ=0.45 (90% CI 0.41-0.48). In terms of validity, the intrarater agreement between PRHISM and subjective assessments by breast experts was κ=0.37 (95% CI 0.14-0.60), while DISCERN showed an agreement of κ=0.27 (95% CI 0.07-0.48), indicating fair agreement and no significant difference in validity.

**Conclusions:**

PRHISM has demonstrated sufficient reliability and validity for evaluating the quality of health-related information on YouTube, making it a promising new metric. To further enhance objectivity, it is necessary to explore the use of artificial intelligence and other approaches.

## Introduction

In recent years, advances in medical technology and diagnostic methods have made health care increasingly complex, leading to a growing tendency for patients to seek information about their disease and treatments [1]. Many patients feel anxious during this process, and internet use for collecting medical information is increasing [2]. In Japan, about 50% of people use the internet as a method for collecting medical information [3]. The sources of information include those officially provided by national cancer centers, as well as those from social media. On social media, anyone can post information regardless of their expertise or qualifications, making it very difficult for patients to judge the quality of that information, which is often a mix of reliable and unreliable sources [4]. The spread of misinformation about vaccines during the COVID-19 pandemic highlighted the problem of inaccurate medical information on social media [5,6]. In response to this issue, the National Academy of Medicine (NAM) published a guide in 2021 to help identify reliable sources of health information on social media [4].

Methods for objectively evaluating the quality of medical information available on the internet have included Diagnostic Information Support Communication Evaluation Report Network (DISCERN) [7]. DISCERN was developed as a tool to assess the quality of written information about treatment choices. It is a metric that evaluates the quality of information using 16 questions, consisting of 15 basic assessments and 1 overall assessment, each scored on a 1 (No) to 5 (Yes) Likert scale. This metric has long been considered reliable and is currently used to assess the quality of breast cancer treatment information available on the internet [8,9]. However, since they were developed before the year 2000 and were not designed with social media in mind, there are concerns that they may be inadequate for evaluating social media [10].

Given the increasing reliance on the internet for health information, it is crucial to ensure that the information available to patients is accurate and reliable. Misinformation can lead to incorrect self-diagnosis, inappropriate treatment choices, and increased anxiety, ultimately affecting patient outcomes and public health. Therefore, there is a pressing need to develop and validate tools specifically designed to evaluate the quality of medical information on social media platforms.

Denniss and colleagues [10] developed the Principles for Health-Related Information on Social Media (PRHISM) tool to evaluate health-related information on social media using a modified Delphi method. This tool assesses 13 principles on a 0 (completely unmet)-4 (completely met) Likert scale and allows nonexperts to evaluate the quality of information, potentially reducing reviewer bias. Compared with existing metrics for evaluating information, PRHISM was specifically designed for social media, offering greater logical validity. Its questions are tailored for social media platforms, with additional considerations for readability and accommodations for vision and hearing impairments. Although PRHISM has not yet been widely used for social media evaluation, its development through appropriate methods and its adaptability suggest it could be valuable for this purpose.

To address these challenges, we adapted a Japanese version of PRHISM and evaluated its reliability and validity. We also conducted assessments using DISCERN, widely used for evaluating medical information quality, and compared the results with PRHISM. Our goal was to evaluate whether PRHISM could adequately assess information quality on Japanese social media and, using this tool, create an environment where patients can access accurate medical information.

## Methods

### Translation of the PRHISM

To use PRHISM in Japan, we obtained permission from the developers of PRHISM. First, PRHISM was translated from English to Japanese by a native Japanese speaker (H Kusama). Then, the Japanese version was back-translated into English by a native English speaker. Any discrepancies between the back-translated English version and the original text were identified, and the Japanese version appropriately adjusted (Multimedia Appendix 1).

### Social Media Platform

The evaluation of PRHISM was conducted using YouTube (Google). YouTube is the second most used platform in Japan after LINE (LY Corporation) and is used across all age groups [11]. Among various platforms, we determined that YouTube is suitable for this evaluation because it allows the posting of longer videos, enabling experts to adequately assess the medical information provided.

### How to Search

Since YouTube uses an algorithm that analyzes viewing history through artificial intelligence (AI) to prioritize related videos, searches were conducted using the internet browser Google Chrome in incognito mode, with no login session active. The search terms used were the Japanese words “乳癌” (にゅうがん, Nyugan, breast cancer), “治療” (ちりょう, Tiryou, treatment), and “抗癌剤” (こうがんざい, Kouganzai, chemotherapy). The search was conducted on January 5, 2024, and the results were sorted by relevance, with the videos listed in order from the top. The exclusion criteria were (1) languages other than Japanese, (2) fewer than 3000 views, (3) shorter than 60 seconds, (4) irrelevant videos, (5) without audio, (6) YouTube shorts, (7) duplicates, and (8) advertisements.

The sources of the videos were categorized into eight categories: (1) health profession schools and other educational institutions (schools of medicine, pharmacy, etc); (2) health care facilities (hospitals, clinics, etc); (3) nonprofit health plans; (4) public health departments (national statement, regional statement, etc); (5) individual health care professionals (doctors, nurses, occupational therapists, etc); (6) entertainment, media, news; (7) personal blogs; and (8) other.

These categories were created with reference to the reliable sources identified by the National Academy of Medicine [4]. Categories 1‐4 were defined as content from reliable sources, while categories 5‐8 were defined as content from other sources. In addition, general information, such as the uploader, number of channel subscribers, number of views, video length, upload date, number of likes, and time since posting, was also collected.

### Evaluators

In total, 6 physicians with expertise in breast cancer conducted the evaluations. All of them were surgical oncologists, and the authors recruited participants from their own institution and affiliated hospitals for this study. : KA (8 y as a physician, 1 y as a breast expert), YI (9 y as a physician, 2 y as a breast expert), RS (10 y as a physician, 2 y as a breast expert), HS (16 y as a physician, 7 y as a breast expert), YH (24 y as a physician, 15 y as a breast expert), and H Kaise (37 y as a physician, 20 y as a breast expert).

### Evaluation Method

In total, 6 physicians with expertise in breast cancer evaluated a common set of 60 videos. For each video, they used PRHISM and DISCERN for evaluation. In addition, the accuracy and potential harm of the information was assessed.

### PRHISM

PRHISM evaluates the quality of health-related information on social media using 13 principles, which are scored on a 0‐4 Likert scale [9].

Since some items may not be applicable depending on the content of the video, the score is calculated based on the applicable questions and converted to a score out of 100 (PRHISM score). Scores of 100‐76 are rated as excellent, 75‐51 as good, 50‐26 as mediocre, and 25‐0 as poor (Textbox 1).

Textbox 1. **Summary of the evaluation tools used in this study.**

**Principles for Health-Related Information on Social Media (PRHISM)**

PRHISM is comprised of 13 principles. Each principle is scored on a 5-point Likert scale (0-4).

Principles:

1. Authorship
2. Authoritative
3. Action-oriented
4. Financial disclosure
5. Attribution
6. Balance and justifiability
7. Risks and benefits
8. Privacy
9. Complementary information
10. Referrals and support
11. Readability and comprehensibility
12. Accessibility
13. Images

**DISCERN**

DISCERN is comprised of 8 reliability assessments, 7 information quality assessments, and one overall quality assessment. Each principle is scored on a 5-point Likert scale (1-5).

Reliability:

1. Are the aims clear?
2. Does it achieve its aims?
3. Is it relevant?
4. Is it clear what sources of information were used to compile the publication (other than the author or producer)?
5. Is it clear when the information used or reported in the publication was produced?
6. How good is the quality of information treatment choices?
7. Is it balanced and unbiased?
8. Does it provide details of additional sources of support and information?

Information quality:

1. Does it refer to areas of uncertainty?
2. Does it describe how each treatment works?
3. Does it describe the benefits of each treatment?
4. Does it describe the risks of each treatment?
5. Does it describe what would happen if no treatment is used?
6. Does it describe how the treatment choices affect overall quality of life?
7. Is it clear that there may be more than one possible treatment choice? Does it provide support for shared decision-making?

Overall evaluation:

1. Overall rating of the publications.

**Cancer Expert Assessment Tool**

Cancer expert assessment tool is comprised of 4 question assessments. Two assessments consist of whether the information is true or false and harmful or not harmful, and 2 review reasons why the evaluation was chosen.

Expert Panel Member Assessment:

1. In your opinion, are the primary medical claims within the article accurate?
  - 5: True, 4: Mostly true, 3: Mixture both True/False, 2: Mostly False, 1: False.
2. If you answered, “Mixture both True/False,” “Mostly False” or “False,” why did you answer this way?
3. In your opinion, could the primary medical claims within the article cause harm?
  - 5: Certainly NOT Harmful, 4: Probably NOT Harmful, 3: Uncertain, 2: Probably Harmful, 1: Certainly Harmful.
4. If you answered, “Uncertain” or “Probably Harmful” or “Certainly Harmful,” why did you answer this way?

### DISCERN

DISCERN evaluates the scientific reliability of medical information related to treatment descriptions and assigns a score [8]. The total score is out of 80 points, with each of the 16 assessments contributing up to 5 points (DISCERN score) (Textbox 1).

### Accuracy and Potential Harm of the Information

The subjective assessment was conducted using an assessment tool developed by Johnson et al [12]. This tool assesses whether the provided medical information is accurate or inaccurate, and if considered inaccurate, the reason is marked in a checkbox. In addition, it evaluates whether the information is harmful or not using the same tool. Accurate information is rated as 1, and inaccurate information as 5, on a Likert scale. Similarly, nonharmful information is rated as 1, and harmful information as 5. To align the scoring methods of PRHISM and DISCERN, in this study, accurate or nonharmful information was rated as 5, and inaccurate or harmful information was rated as 1 (Textbox 1).

### Training and Protocol for Evaluators

Each evaluator received a lecture once on how to use the metrics during a preliminary meeting. The principal investigator, who is also the first author of the study, conducted the lecture in an internet-based group session in a group session for approximately 60 minutes. Subsequently, evaluation sheets, along with Japanese translations of the PRHISM and DISCERN guides [10,13], were provided. The evaluators conducted their assessments based on these guides. No further training were conducted thereafter.

Discussion among evaluators regarding each evaluation was not permitted.

### Reliability

Reliability was assessed by examining the agreement of scores between evaluators. In the previous literature on DISCERN, evaluations were based on the agreement of the overall quality score. However, PRHISM does not have a corresponding criterion. For the purpose of statistical evaluation and comparison in this study, we added a “PRHISM overall quality” component, similar to that in DISCERN, which assessed the entire video after evaluating the 13 principles. This component was also scored on a 0‐4 Likert scale, and its agreement was evaluated.

### Validity

There is no gold standard for evaluating the validity of the quality of medical information. For validity, the subjective assessment of the quality of medical information by physicians with expertise in breast cancer was considered an appropriate assessment, and this was used as the standard for validity. Validity was assessed by examining the agreement between the PRHISM overall quality and the experts’ subjective assessments. The validity of DISCERN was also examined in the same way.

### Sample Size Determination

The number of videos to be evaluated was determined based on the interrater agreement for PRHISM overall quality. As there are no reports examining the level of interrater agreement for PRHISM, DISCERN was used as a reference. Currently, DISCERN is the primary tool used to evaluate the quality of medical information on social media. DISCERN assesses 15 criteria and then evaluates the overall quality of the information. In previous studies, the agreement on the overall quality has been evaluated. Cohen kappa was used to evaluate the degree of agreement. The degree of agreement varies depending on the expertise of the evaluators, with a kappa of 0.23 reported for self-help group members, 0.40 for information providers, and 0.53 for an expert [7].

The expected agreement for PRHISM overall quality was assumed to have a threshold of κ=0.53 and an expected value of κ=0.61. The threshold of 0.53 was determined based on the assumption that PRHISM, being a tool specifically designed for evaluating social media, would achieve a higher level of agreement than DISCERN for an expert. The expected value of 0.61 is generally considered to indicate “sufficient agreement” in terms of the kappa coefficient [14]. For the number of videos to be evaluated in this study, it was necessary to assume the distribution of the overall quality scores in PRHISM. Therefore, as a pilot test, the investigator (HK [Hiroki Kusama]) evaluated 50 videos to establish this distribution in Multimedia Appendix 2. Based on the above settings, the number of videos was determined through a simulation experiment conducted 10,000 times. Assuming an alternative hypothesis kappa coefficient of 0.61, the number of videos required to reject the null hypothesis of 0.53 with over 80% power at a 2-sided significance level of 10% was calculated to be 55 for 6 evaluators. The *z* test, approximated by a normal distribution, was used to calculate the test statistics [15]. Anticipating that some videos might be difficult to evaluate, we decided to have 6 evaluators assess 60 videos. The 6 evaluators were distributed, with 3 physicians having more than 10 years of experience and 3 physicians having less than 10 years of experience.

### Statistical Analysis

The primary analysis focused on examining the interrater agreement for PRHISM overall quality (reliability of PRHISM) and DISCERN overall quality (reliability of DISCERN).

As secondary analyses, we examined the following:

- The intrarater agreement between PRHISM overall quality and DISCERN overall quality.
- The intrarater agreement between PRHISM overall quality and expert evaluations (validity of PRHISM).
- The intrarater agreement between DISCERN overall quality and expert evaluations (validity of DISCERN).
- The interrater agreement for PRHISM score and its categories (reliability of PRHISM).
- The interrater agreement for DISCERN score and its categories (reliability of DISCERN).

To compare the DISCERN score with the PRHISM score, we calculated the modified DISCERN score by subtracting the DISCERN overall quality score from the total DISCERN score, dividing the result by the maximum possible score based on the number of applicable questions, and then converting it to a score out of 100 (modified DISCERN score).

The agreement of evaluations was assessed using the kappa coefficient. The interpretation of agreement levels is as follows: <0.00 (no agreement); 0.00‐0.20 (slight); 0.21‐0.40 (fair); 0.41‐0.60 (moderate); 0.61‐0.80 (substantial); and 0.81‐1.00 (almost perfect) [14]. The interobserver agreement was calculated using the kappa coefficient and a 90% CI, and an agreement level of 0.61 or higher was interpreted as sufficient.

For the interrater agreement of the PRHISM overall quality, 2 evaluators were selected from a group of 6, and the agreement for 15 different patterns was calculated. The average and the 90% CI were calculated [15]. The 90% CI was calculated using normal approximation. If the lower limit of the 90% CI exceeded 0.53, the primary analysis (PRHISM reliability) was considered to have been achieved.

For the secondary analyses, the kappa coefficient and its 95% CI were calculated for each pair of evaluators to assess intrarater agreement. Secondary analyses for the interrater agreement were performed using the same approach as the primary analysis.

In addition, since the PRHISM score and the modified DISCERN score are continuous variables, we also performed an analysis using intraclass correlation coefficient. Statistical analyses were performed using R software (version 4.2.3; R Core Team).

### Ethical Considerations

The study was approved by the Institutional Review Board of Tokyo Medical University (T2024-0034). The study involved an analysis of publicly available YouTube data, which does not require individual consent from participants. However, ethical approval was obtained to ensure that the research adhered to institutional guidelines for research involving public data. No compensation was provided to participants as this study involved an analysis of publicly available data. Should any concerns or complaints be raised by video contributors or their families regarding ethical or social issues, the principal investigator will respond sincerely and appropriately in line with institutional procedures.

## Results

### Overview

**Figure 1.**
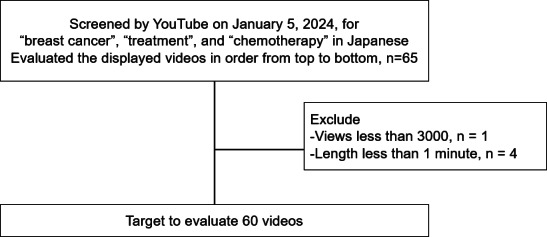
Flowchart of video selection in this study. In total, 60 videos were selected after excluding those with fewer views or short duration.

Using the predefined search method, we excluded 5 videos in total (1 video with fewer than 3000 views and 4 videos with a duration of less than 1 minute), resulting in a list of 60 videos. A CONSORT (Consolidated Standards of Reporting Trials) diagram is shown in Figure 1.

The median video length was 8 (range: 1‐126) minutes, the median number of views was 30,542.5 (range: 3921‐978,676), and the median time since posting was 29 (range: 7‐123) months. The sources of the videos were as follows: 5 individual health care professionals accounted for the largest category with 15 videos (25%); followed by 6 entertainment, media, and news with 13 videos (22%); 7 personal blogs with 12 videos (20%); 3 nonprofit health plans with 9 videos (15%); 1 health professions schools and other educational institutions with 6 videos (10%); 2 health care organizations with 4 videos (7%); and 8 other sources with 1 video (2%). No videos were posted from the public health departments (Table 1).

**Table 1.** The characteristics of selected videos on YouTube.

Characteristics		Statistical value (n=60)
Video length (mins), median (range)		8 (1-126)
Views, n (range)		30,542.5 (3921-978,676)
Time since posting (months), median (range)		29 (7-123)
Sources of videos, n (%)		
	Health professions schools and other educational institutions (eg, medical schools and pharmacy schools)	6 (10)
	Health care organizations (eg, academic medical centers and specialty hospitals)	4 (7)
	Nonprofit health plans	9 (15)
	Public health departments	0 (0)
	Individual health care professionals	15 (25)
	Entertainment and media news	13 (22)
	Breast cancer survivor’s blog	12 (20)
	Others	1 (2)

### Primary Analysis

#### Reliability

**Figure 2.**
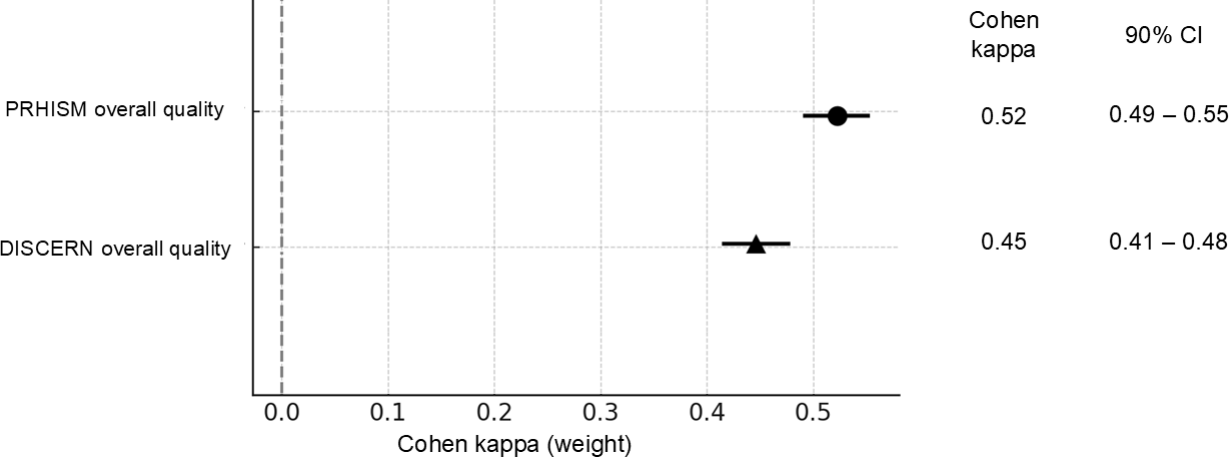
Interrater agreement on PRHISM and DISCERN overall quality. The circle and triangle represent the mean kappa and CIs (90% CI) are represented by horizontal error bars. DISCERN: Diagnostic Information Support Communication Evaluation Report Network; PRHISM: Principles for Health-Related Information on Social Media.

The interrater agreement for PRHISM overall quality was κ=0.52 (90% CI 0.49-0.55), indicating moderate agreement. Since the lower limit of the 90% CI was below 0.53, the primary analysis was not achieved. However, the interrater agreement for DISCERN overall quality was κ=0.45 (90% CI 0.41-0.48), also indicating moderate agreement. The 90% CI did not overlap, suggesting that PRHISM may be a superior measure in terms of interrater agreement compared with DISCERN (Figures2  3).

**Figure 3.**
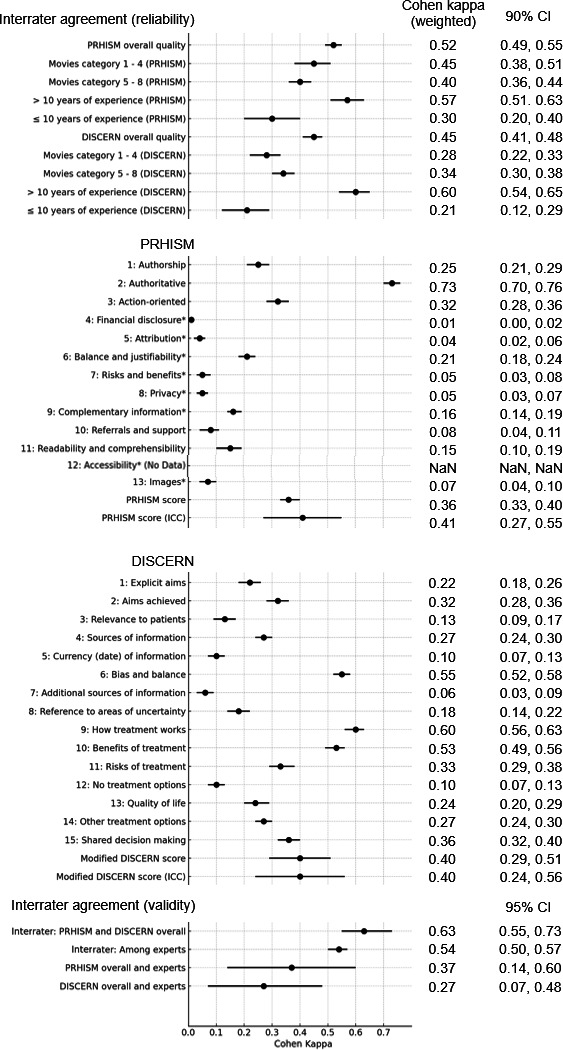
Results of reliability and validity agreement. The circle represents the mean kappa and CIs (90 or 95% CI) are represented by horizontal error bars. DISCERN: Diagnostic Information Support Communication Evaluation Report Network; ICC: intraclass correlation coefficients; NaN: not a number; PRHISM: Principles for Health-Related Information on Social Media.

#### Subgroup Analysis in Primary Analysis

For videos originating from reliable sources (categories 1‐4), the PRHISM overall quality was κ=0.45 (90% CI 0.38-0.51), and the DISCERN overall quality was κ=0.28 (90% CI 0.22-0.33). For videos from other sources (categories 5‐8), the PRHISM overall quality was κ=0.40 (90% CI 0.36-0.44), and the DISCERN overall quality was κ=0.34 (90% CI 0.30-0.38; Figure 3, Multimedia Appendix 3). The 90% CI for PRHISM from reliable sources did not overlap with the 90% CI for DISCERN.

For those with over 10 years of experience, the PRHISM overall quality was κ=0.57 (90% CI 0.51-0.63), and the DISCERN overall quality was κ=0.60 (90% CI 0.54-0.65). For those with less than 10 years of experience, the PRHISM overall quality was κ=0.30 (90% CI 0.20-0.40), and the DISCERN overall quality was κ=0.21 (90% CI: 0.12-0.29). When the years of experience were 10 or more, the agreement on overall quality was higher for both PRHISM and DISCERN compared with those with less than 10 years of experience (Figure 3, Multimedia Appendix 4).

### Secondary Analysis

Intrarater agreement for PRHISM overall quality and DISCERN overall quality was κ=0.63 (95% CI 0.55-0.73), indicating substantial agreement. In evaluating medical information on social media, there was no significant difference between the assessments of PRHISM overall quality and DISCERN overall quality (Figure 3).

The intrarater agreement among experts was κ=0.54 (95% CI 0.50-0.57), indicating moderate agreement (Figure 3).

### Validity

We evaluated the agreement between the PRHISM overall quality and DISCERN overall quality scores with the quality of information as subjectively assessed by experts. The agreement between PRHISM overall quality and the experts’ subjective assessment was κ=0.37 (95% CI 0.14-0.60), indicating fair agreement. The agreement between DISCERN overall quality and the experts’ subjective assessment was κ=0.27 (95% CI 0.07-0.48), indicating fair agreement. The 95% CIs overlapped, suggesting that the validity was considered equivalent (Figures3  4). The 95% CIs for the agreement of each of the 6 evaluators all overlapped, but the level of agreement varied among specialists for both PRHISM and DISCERN, ranging from κ=0.07 to 0.65 and κ=0.01 to 0.51, respectively. (Multimedia Appendix 5).

The circle represents the mean kappa for PRHISM, and triangle represent the mean kappa for DISCERN. The 95% CIs are represented by horizontal error bars.

**Figure 4.**
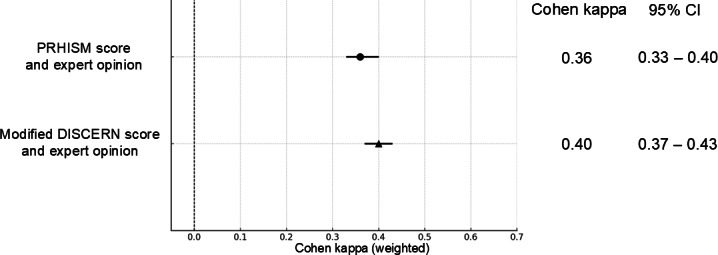
Intrarater agreement between PRHISM or DISCERN overall quality and expert opinion, the circle and triangle represent the mean kappa and CIs (95% CI) are represented by horizontal error bars. DISCERN: Diagnostic Information Support Communication Evaluation Report Network; PRHISM: Principles for Health-Related Information on Social Media.

### Agreement Between PRHISM Score and DISCERN Score

**Figure 5.**
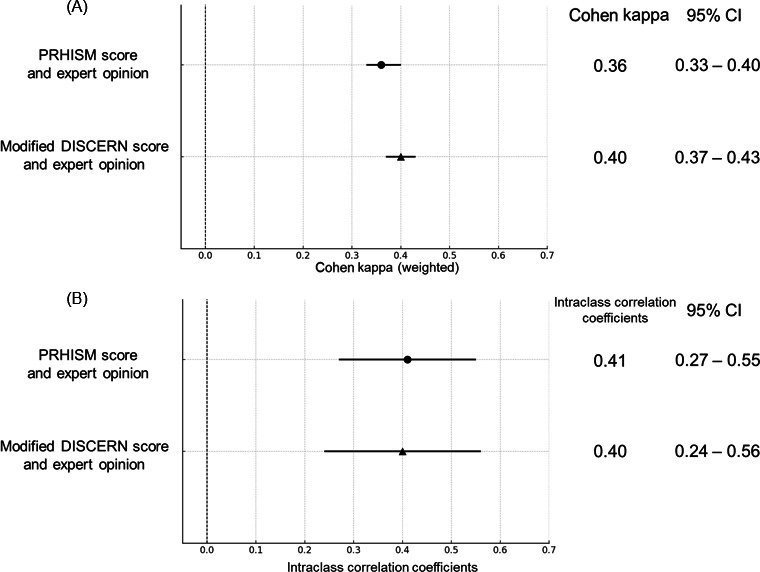
Intra-rater agreement between PRHISM score and modified DISCERN score and expert opinion. The circle and triangle represents the mean kappa and 95% CIs are represented by horizontal error bars. (**A**) Cohen kappa. (**B**) Intraclass correlation coefficient. DISCERN: Diagnostic Information Support Communication Evaluation Report Network; PRHISM: Principles for Health-Related Information on Social Media.

We evaluated the interrater agreement among 6 evaluators for the PRHISM score and the modified DISCERN score, both rated on a score out of 100. The interrater agreement for the PRHISM score and the modified DISCERN score was κ=0.36 (95% CI 0.33-0.40) and κ=0.40 (95% CI 0.37-0.43), respectively (Figure 5A). Using the intraclass correlation coefficient, the PRHISM score was 0.41 (95% CI 0.27-0.55) and the DISCERN score was 0.40 (95% CI 0.24-0.56) (Figure 5B).

The circle represents the mean kappa for PRHISM, and the triangle represent the mean kappa for DISCERN. The 95% CIs are represented by horizontal error bars.

### Agreement for Each Evaluation Question of PRHISM and DISCERN

For PRHISM, when a question was judged as “not applicable,” it was excluded from the score calculation. Therefore, some were assessed using unweighted Cohen kappa. The highest agreement was for question 2, “authoritative,” with κ=0.73 (95% CI 0.70-0.76). For DISCERN, the highest agreement was for item 12, “benefits of treatment,” with κ=0.60 (95% CI 0.56-0.63) (Figure 3).

In addition, PRHISM is a metric that classifies video quality as poor, mediocre, good, or excellent based on the PRHISM score. We evaluated the agreement between the subjective assessments by experts and the PRHISM scoring classification. When assigning 1 to poor and 4 to excellent, the agreement was κ=0.54 (95% CI 0.45-0.64), indicating moderate agreement (Multimedia Appendix 6).

### Evaluation of the Quality of Breast Cancer Treatment Information on YouTube in Japan Using PRHISM and DISCERN Score

Although this study primarily examined the utility of PRHISM, we also evaluated the quality of breast cancer treatment information on YouTube in Japan using PRHISM and DISCERN scores. The mean PRHISM and DISCERN scores for medical information related to breast cancer treatment on Japanese YouTube were 60.6 (SD 11.5) and 58.9 (SD 11.5), respectively (Multimedia Appendix 7).

## Discussion

### Principal Findings

In evaluating the quality of medical information on YouTube, the interrater agreement for the overall quality score of PRHISM was κ=0.52 (90% CI 0.49-0.55), and the primary end point was not achieved. However, the 90% CI for interrater agreement of PRHISM was superior to that of DISCERN, indicating that PRHISM is a more reliable metric when evaluating the quality of medical information on YouTube. In terms of validity, the agreement between the experts’ subjective assessment and PRHISM overall quality was κ=0.37 (95% CI 0.14-0.60), indicating fair agreement. It was found that PRHISM has validity equivalent to that of DISCERN.

Although the primary end point was not achieved, 1 possible reason was the difficulty in setting the threshold and expected values for interrater agreement of PRHISM. In setting the threshold and expected values, we used DISCERN as a reference because no studies have examined the interrater agreement of PRHISM [7,10]. While the number of evaluators and videos was determined statistically, it is possible that a larger number of both was necessary to adequately evaluate the quality of information. However, DISCERN was designed for books available in public libraries and bookstores, and leaflets produced by professional organizations and national self-help groups. We referred to studies that used DISCERN to evaluate medical information on social media, but many of them used a modified DISCERN with fewer evaluation items, making them difficult to reference [16-22]. DISCERN was not developed for social media, so the agreement may differ from previous studies. In fact, the agreement for DISCERN in this study, which focused on social media, was 0.45, lower than the 0.53 reported in previous studies [7] , suggesting that the threshold and expected values might have been better set slightly lower. Therefore, given the results obtained with this threshold, no definitive conclusion can be drawn about the robustness of PRHISM.

In previous studies, the agreement for DISCERN decreased depending on the profession of the evaluators [7]. In this study as well, a difference in agreement was observed depending on whether the years of experience were 10 or more, or less than 10. Although PRHISM is designed to allow for evaluation by nonexperts, these results suggest that evaluations conducted by experts may be more accurate.

We also examined validity. However, there is no gold standard for evaluating the quality of medical information. Therefore, it is necessary to establish a consensus among experts. During the development of PRHISM, the modified Delphi method, a consensus-building technique, was used. In addition, we assessed validity by setting alternative criteria. In previous reports, some studies have assessed validity based on guidelines and evidence [23] , while others have used expert evaluations as the standard [12,24] . In addition, there are studies that have used DISCERN as an alternative criterion. In this study, using expert evaluations as the standard, the agreement among experts was κ=0.54 (95% CI 0.50-0.57), showing a moderate level of consistency. However, the agreement between the experts’ evaluations and PRHISM overall quality was κ=0.37 (95% CI 0.14-0.60), showing only fair agreement. Nevertheless, since the agreement was comparable with that of DISCERN, it suggests that PRHISM is also sufficiently valid for evaluating the quality of information. The lack of strong agreement may be due to the inevitable subjectivity of the assessments, leading to variations in judgments based on each expert’s preferences and perspectives. In fact, the level of agreement varied among experts (Multimedia Appendix 5). It can be considered to have at least comparable validity to DISCERN, but there may be a need to consider how to use this tool regarding its validity.

### Future Prospects

We aim to explore the use of AI to make the evaluation of medical information more objective, efficient, and with higher validity, allowing for the assessment of a larger volume of information in a shorter time. In fact, there are reports investigating whether AI can be used to evaluate online health information [25] . This study demonstrated that PRHISM is a suitable tool for evaluating the quality of medical information on social media. Therefore, this research serves as an important first step toward further investigations using PRHISM to assess the quality of medical information in various social media contexts. If low-quality information could automatically trigger warnings, it would help ensure that patients receive higher-quality medical information. Future research will explore the extent to which AI can be integrated into the evaluation process.

### Limitations

This study has some limitations. First, the PRHISM overall quality is a metric independently established by the authors and was created specifically for statistical analysis. Since PRHISM is a tool that evaluates information using the PRHISM score or PRHISM scoring classification [10], the study may not directly evaluate the tool itself.

Second, there was a small sample size and a limited number of evaluators. Although a statistically valid number was considered, there was variability in the evaluations among the experts, potentially influenced by differences in their years of clinical experience. This indicates that a larger sample size and a more diverse group of evaluators with varying levels of expertise might be needed. To address this issue, future studies could include standardized training modules to improve consistency. In addition, integrating AI could automate certain aspects of scoring, reducing human bias and increasing efficiency.

Third, this study presents results solely from Japan. There may be an influence on the results due to biases in breast cancer treatment practices and expertise in Japan, as well as differences in medical environments. In addition, this study was limited to information about breast cancer treatment on YouTube, and the findings may not be applicable to other diseases or health-related information on different social media platforms. Similar studies need to be conducted in other countries and on different social media platforms. Although YouTube has regulations on posted videos, some reports indicate that the quality of health-related videos on YouTube varies widely, from low to high. Therefore, further research is needed to determine whether PRHISM is effective for evaluating content in other countries or on different social media platforms, as this will enhance its utility and relevance globally. We plan to conduct evaluations on other social media platforms.

### Conclusions

PRHISM has greater reliability than DISCERN in evaluating the quality of medical information on social media, with comparable validity. It has the potential to become a standard metric for assessing the quality of medical information on social media.

## Supplementary material

10.2196/66416 Multimedia Appendix 1 PRHISM English translation. PRHISM: Principles for Health-Related Information on Social Media.

10.2196/66416 Multimedia Appendix 2 Result of pilot study. The results of an evaluation of the top 50 YouTube videos searched by the representative (H Kusama). The search terms, search method, and exclusion criteria are the same as those used in this study. The y-axis indicates the number of videos, while the x-axis represents the PRHISM overall quality score. PRHISM: Principles for Health-Related Information on Social Media.

10.2196/66416 Multimedia Appendix 3 Intrarater agreement between PRHISM/DISCERN overall quality and each expert opinion. The y-axis displays the initials of the breast experts who made the assessments. The circle represents the mean kappa for PRHISM, and the triangle represent the mean kappa for DISCERN. The 95% CIs are represented by horizontal error bars. DISCERN: Diagnostic Information Support Communication Evaluation Report Network; PRHISM: Principles for Health-Related Information on Social Media.

10.2196/66416 Multimedia Appendix 4 Subgroup analysis of interrater agreement on PRHISM and DISCERN. The y-axis displays the initials of the breast experts, divided by experience level (over 10 years and 10 years or less). The circle represents the mean kappa for PRHISM, and the triangle represent the mean kappa for DISCERN. The 95% CIs are represented by horizontal error bars. DISCERN: Diagnostic Information Support Communication Evaluation Report Network; PRHISM: Principles for Health-Related Information on Social Media.

10.2196/66416 Multimedia Appendix 5 Subgroup analysis of interrater agreement on PRHISM and DISCERN. The y-axis displays the initials of the breast experts, divided by video categories (1–4 and 5–8). The circle represents the mean kappa for PRHISM, and the triangle represent the mean kappa for DISCERN. The 95% CIs are represented by horizontal error bars. DISCERN: Diagnostic Information Support Communication Evaluation Report Network; PRHISM: Principles for Health-Related Information on Social Media.

10.2196/66416 Multimedia Appendix 6 Intrarater agreement between PRHISM score classification and expert opinion. The y-axis displays the initials of the breast experts who made the assessments. The 95% CIs are represented by horizontal error bars. PRHISM: Principles for Health-Related Information on Social Media.

10.2196/66416 Multimedia Appendix 7 PRHISM and DISCERN Scores for evaluating medical information on Japanese YouTube. Box plots showing the distribution of PRHISM and DISCERN scores for medical information related to breast cancer treatment on Japanese YouTube. The boxes represent the IQR, with the horizontal line indicating the median. The vertical extending lines shows the minimum and maximum values within 1.5 times the IQR. DISCERN: Diagnostic Information Support Communication Evaluation Report Network; PRHISM: Principles for Health-Related Information on Social Media.

## Abbreviations

AI: artificial intelligence
CONSORT: Consolidated Standards of Reporting Trials
DISCERN: Diagnostic Information Support Communication Evaluation Report Network
PRHISM: Principles for Health-Related Information on Social Media
