# Supplementary material for: Assessing the Reliability and Validity of Principles for Health-Related Information on Social Media (PRHISM) for Evaluating Breast Cancer Treatment Videos on YouTube: Instrument Validation Study
Source: JMIR Infodemiology. 2025 Jun 11;5:e66416. doi: 10.2196/66416 (PMC12175871; doi:10.2196/66416)
Supplement: Multimedia Appendix 1 [file infodemiology-v5-e66416-s001.docx]

PRHISM Scoring Tool

| **Principle 1: Information of the Author (Authorship)**  *When providing health-related information on social media, the names, credentials, affiliations, and contact information of authors and contributors should be clearly stated on their social media profiles.* | | | | | | | | | | | |
| --- | --- | --- | --- | --- | --- | --- | --- | --- | --- | --- | --- |
| 0 | 1 | | 2 | | 3 | | 4 | | ***Score*** | | ***Possible Score*** |
| Completely noncompliant | Partially compliant | | | | | | Completely compliant | |  | |  |
| **Principle 2: Qualification (Authoritative)**  *Health-related information on social media should be provided by qualified experts in health science and medicine, and the information should only be within the scope of the author's or contributor's qualifications. If information is provided by an unqualified person, it should be clearly stated.* | | | | | | | | | | | |
| 0 | 1 | | 2 | | 3 | | 4 | | ***Score*** | | ***Possible Score*** |
| Completely noncompliant | Partially compliant | | | | | | Completely compliant | |  | |  |
| **Principle 3: Content to Encourage Action (Action-oriented)**  *Health-related information provided on social media should be a call to action for consumers. It should include clear and concise messages that support decision-making and provide context for the information.* | | | | | | | | | | | |
| 0 | 1 | | 2 | | 3 | | 4 | | ***Score*** | | ***Possible Score*** |
| Completely noncompliant | Partially compliant | | | | | | Completely compliant | |  | |  |
| **Principle 4: Financial Disclosure**  *The existence of sponsorship, advertising, funding, financial support, and potential conflicts of interest should be clearly stated.* | | | | | | | | | | | |
| 0 | 1 | | 2 | | 3 | | 4 | | ***Score*** | | ***Possible Score*** |
| Completely noncompliant | Partially compliant | | | | | | Completely compliant | |  | |  |
| **Principle 5: Information Source (Attribution)**  *Health-related information on social media used for the post should clearly state the source of the information and include a hyperlink.* | | | | | | | | | | | |
| 0 | 1 | | 2 | | 3 | | 4 | | ***Score*** | | ***Possible Score*** |
| Completely noncompliant | Partially compliant | | | | | | Completely compliant | |  | |  |
| **Principle 6: Balance & Justifiability of Information**  *When health-related information on social media includes claims about the benefit or performance of a particular treatment, product, service, or health behavior, it should be balanced, unbiased, and supported by appropriate, high-quality evidence.* | | | | | | | | | | | |
| 0 | 1 | 2 | | 3 | | 4 | | ***Score*** | | ***Possible Score*** | |
| Completely noncompliant | Partially compliant | | | | | Completely compliant | |  | |  | |
| **Principle 7: Risks & Benefits**  *When providing health-related information on social media, a summary of the risks and benefits associated with a particular treatment, product, service, or health behavior should be clearly outlined.* | | | | | | | | | | | |
| 0 | 1 | 2 | | 3 | | 4 | | ***Score*** | | ***Possible Score*** | |
| Completely noncompliant | Partially compliant | | | | | Completely compliant | |  | |  | |
| **Principle 8: Privacy**  *The principles of privacy and confidentiality should be respected for health-related information on social media.* | | | | | | | | | | | |
| 0 | 1 | 2 | | 3 | | 4 | | ***Score*** | | ***Possible Score*** | |
| Completely noncompliant | Partially compliant | | | | | Completely compliant | |  | |  | |
| **Principle 9: Complementary Information**  *Health-related information on social media should support, but not replace, your relationship with your physician and other healthcare providers. Posts must emphasize discussing health-related information with consumers’ healthcare providers.* | | | | | | | | | | | |
| 0 | 1 | 2 | | 3 | | 4 | | ***Score*** | | ***Possible Score*** | |
| Completely noncompliant | Partially compliant | | | | | Completely compliant | |  | |  | |
| **Principle 10: Referrals & Support**  *Health-related information on social media should include referrals to (or mentions of) further sources of support and information.* | | | | | | | | | | | |
| 0 | 1 | 2 | | 3 | | 4 | | ***Score*** | | ***Possible Score*** | |
| Completely noncompliant | Partially compliant | | | | | Completely compliant | |  | |  | |
| **Principle 11: Readability & Comprehensibility**  *When providing health-related information on social media, jargon and medical terms that are difficult to understand should be avoided. Plain language should be used for easy understanding by the general public.* | | | | | | | | | | | |
| 0 | 1 | 2 | | 3 | | 4 | | ***Score*** | | ***Possible Score*** | |
| Completely noncompliant | Partially compliant | | | | | Completely compliant | |  | |  | |
| **Principle 12: Accessibility**  *Health-related information on social media should be accessible to people with visual and hearing impairments.* | | | | | | | | | | | |
| 0 | 1 | 2 | | 3 | | 4 | | ***Score*** | | ***Possible Score*** | |
| Completely noncompliant | Partially compliant | | | | | Completely compliant | |  | |  | |
| **Principle 13: Images**  *When images are included in health-related posts on social media, they should be visually clear and not be inconsistent with the information in the post.* | | | | | | | | | | | |
| 0 | 1 | 2 | | 3 | | 4 | | ***Score*** | | ***Possible Score*** | |
| Completely noncompliant | Partially compliant | | | | | Completely compliant | |  | |  | |

|  | ***Score*** | ***Possible Score*** |
| --- | --- | --- |
| Total score |  |  |
| Total score/possible score × 100 = PRHISM score  PRHISM Score: |  | |
