# Supplementary figures and images for: Assessing the Reliability and Validity of Principles for Health-Related Information on Social Media (PRHISM) for Evaluating Breast Cancer Treatment Videos on YouTube: Instrument Validation Study
Source: JMIR Infodemiology. 2025 Jun 11;5:e66416. doi: 10.2196/66416 (PMC12175871; doi:10.2196/66416)

## Slide 1
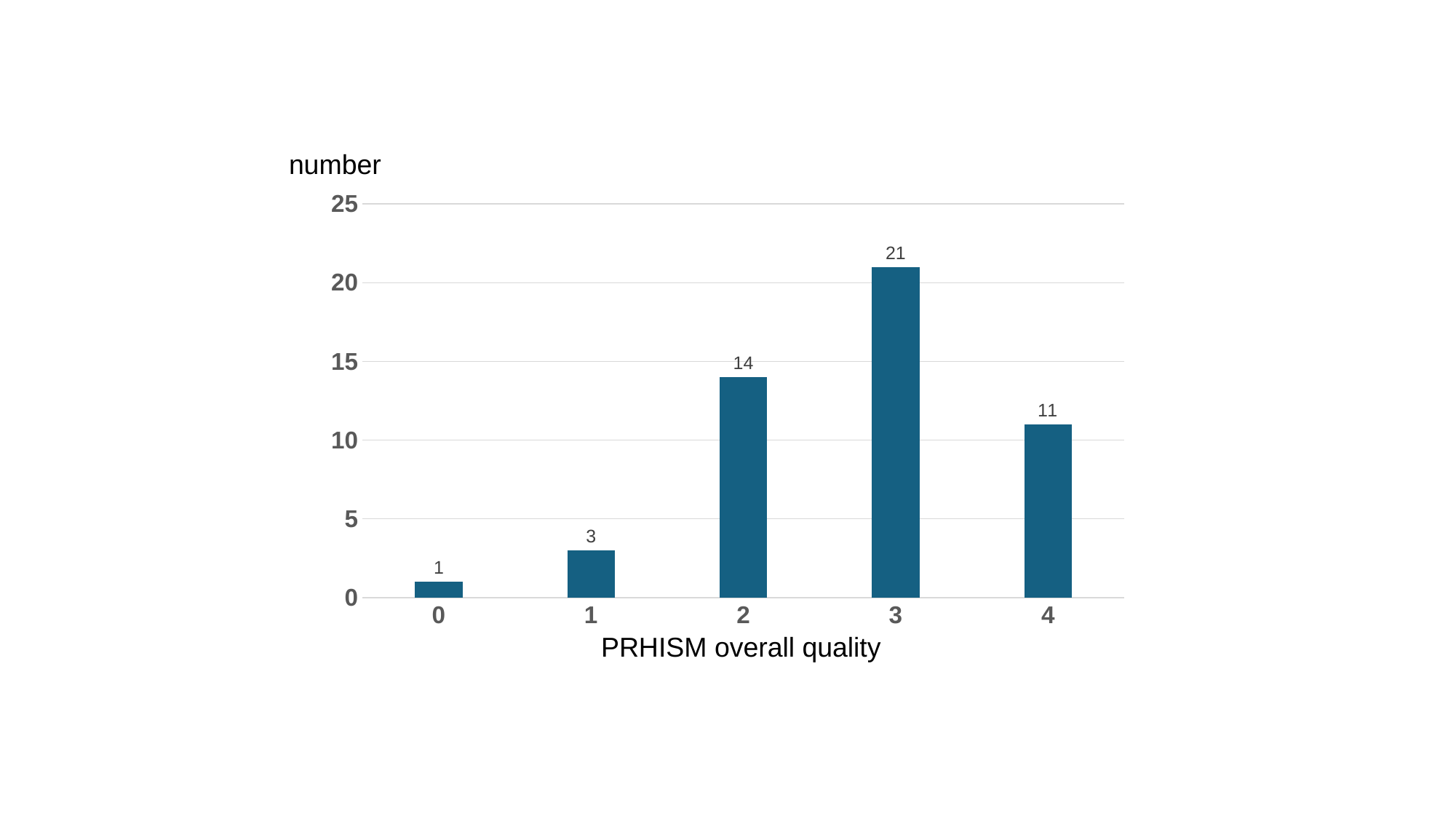

number
### Chart
| Category | number |
|---|---|
| 0 | 1.0 |
| 1 | 3.0 |
| 2 | 14.0 |
| 3 | 21.0 |
| 4 | 11.0 |PRHISM overall quality

Supplement: Multimedia Appendix 2 [file infodemiology-v5-e66416-s002.pptx]

## Slide 1
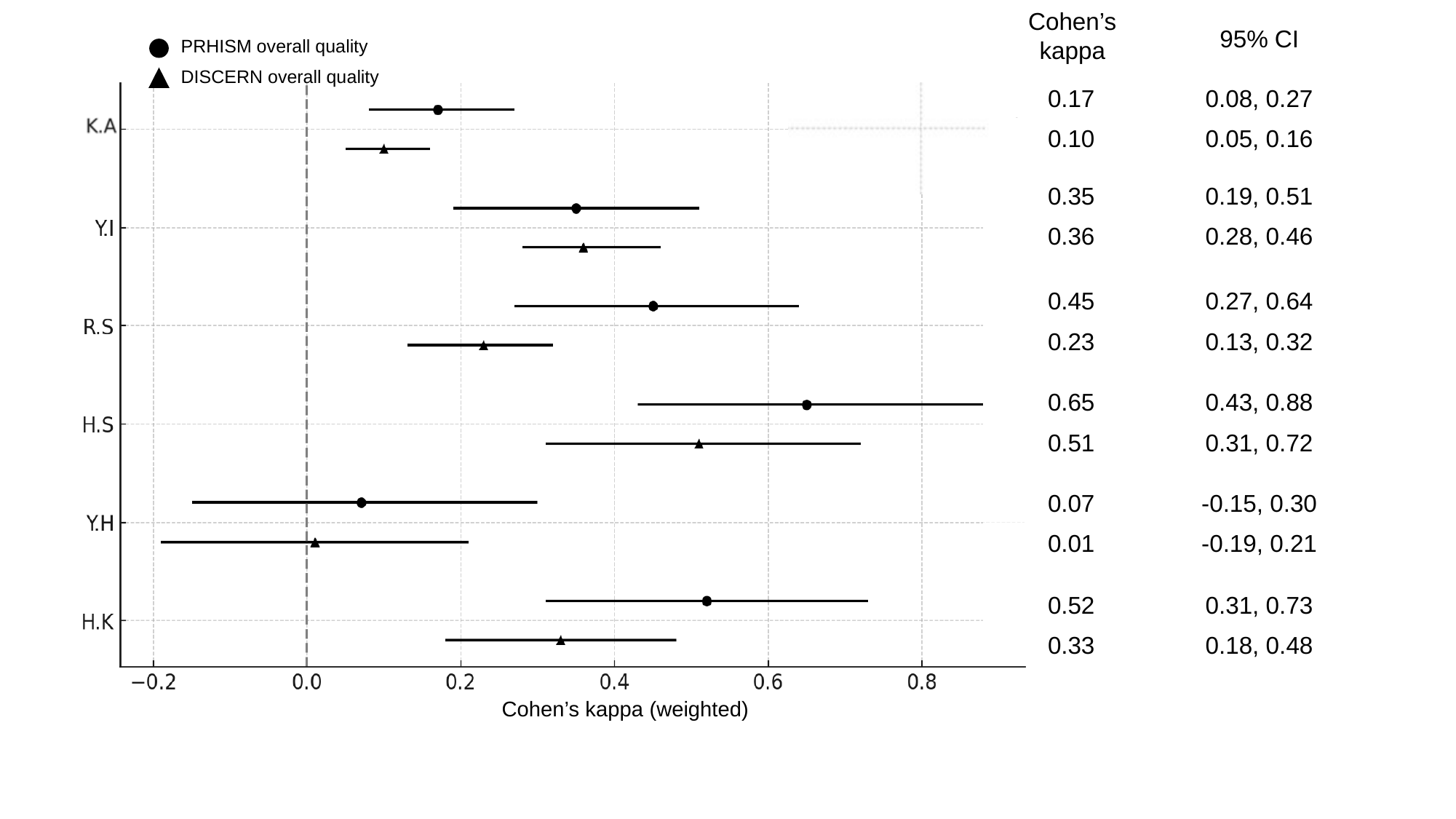

Cohen’s
kappa
95% CI
PRHISM overall quality
DISCERN overall quality
0.17
0.08, 0.27
0.10
0.05, 0.16
0.35
0.19, 0.51
0.36
0.28, 0.46
0.45
0.27, 0.64
0.23
0.13, 0.32
0.65
0.43, 0.88
0.51
0.31, 0.72
0.07
-0.15, 0.30
0.01
-0.19, 0.21
0.52
0.31, 0.73
0.33
0.18, 0.48
Cohen’s kappa (weighted)

Supplement: Multimedia Appendix 3 [file infodemiology-v5-e66416-s003.pptx]

## Slide 1
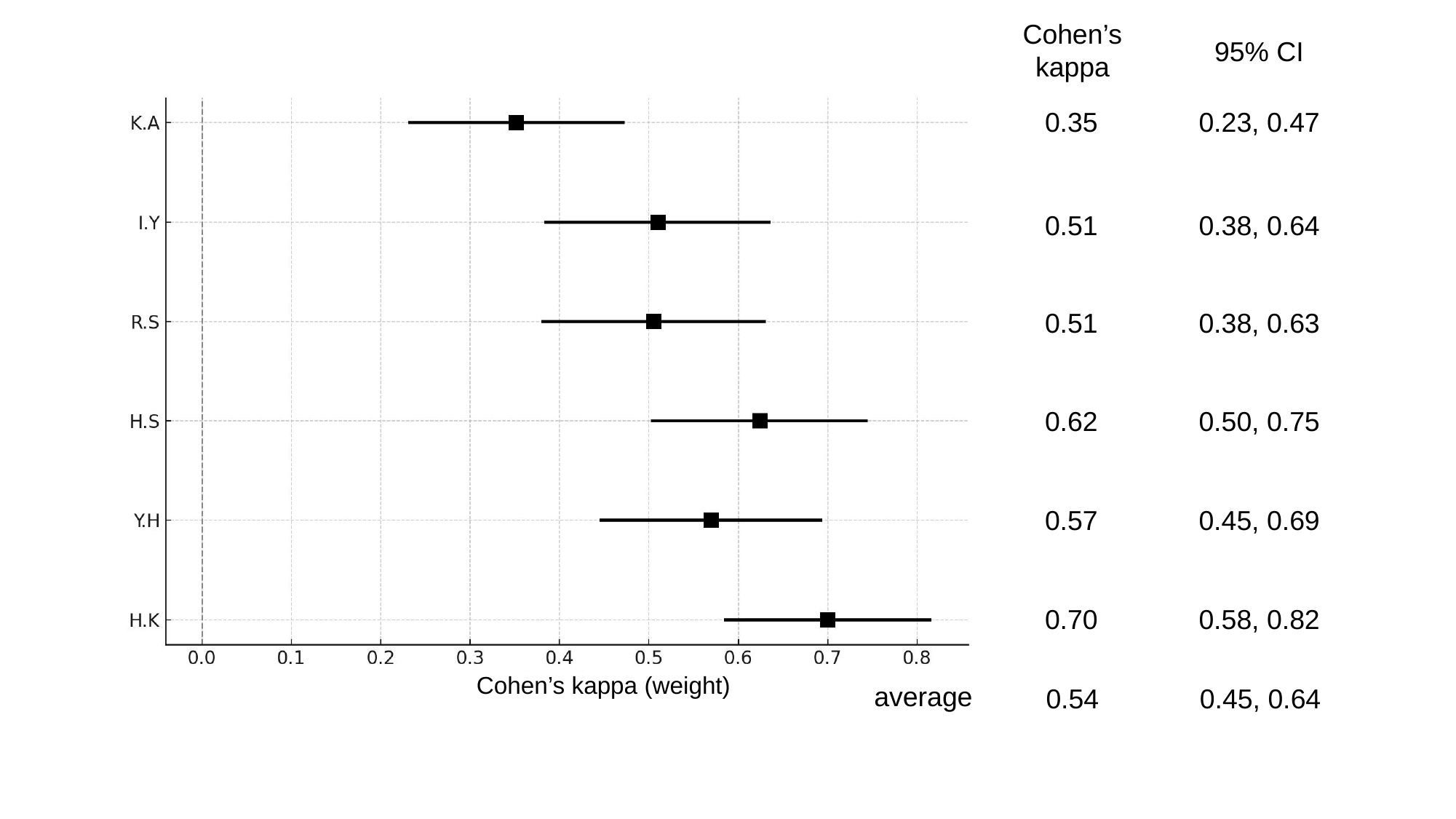

Cohen’s
kappa
95% CI
0.35
0.23, 0.47
0.51
0.38, 0.64
0.51
0.38, 0.63
0.62
0.50, 0.75
0.57
0.45, 0.69
0.70
0.58, 0.82
Cohen’s kappa (weight)
average
0.54
0.45, 0.64

Supplement: Multimedia Appendix 6 [file infodemiology-v5-e66416-s006.pptx]

## Slide 1
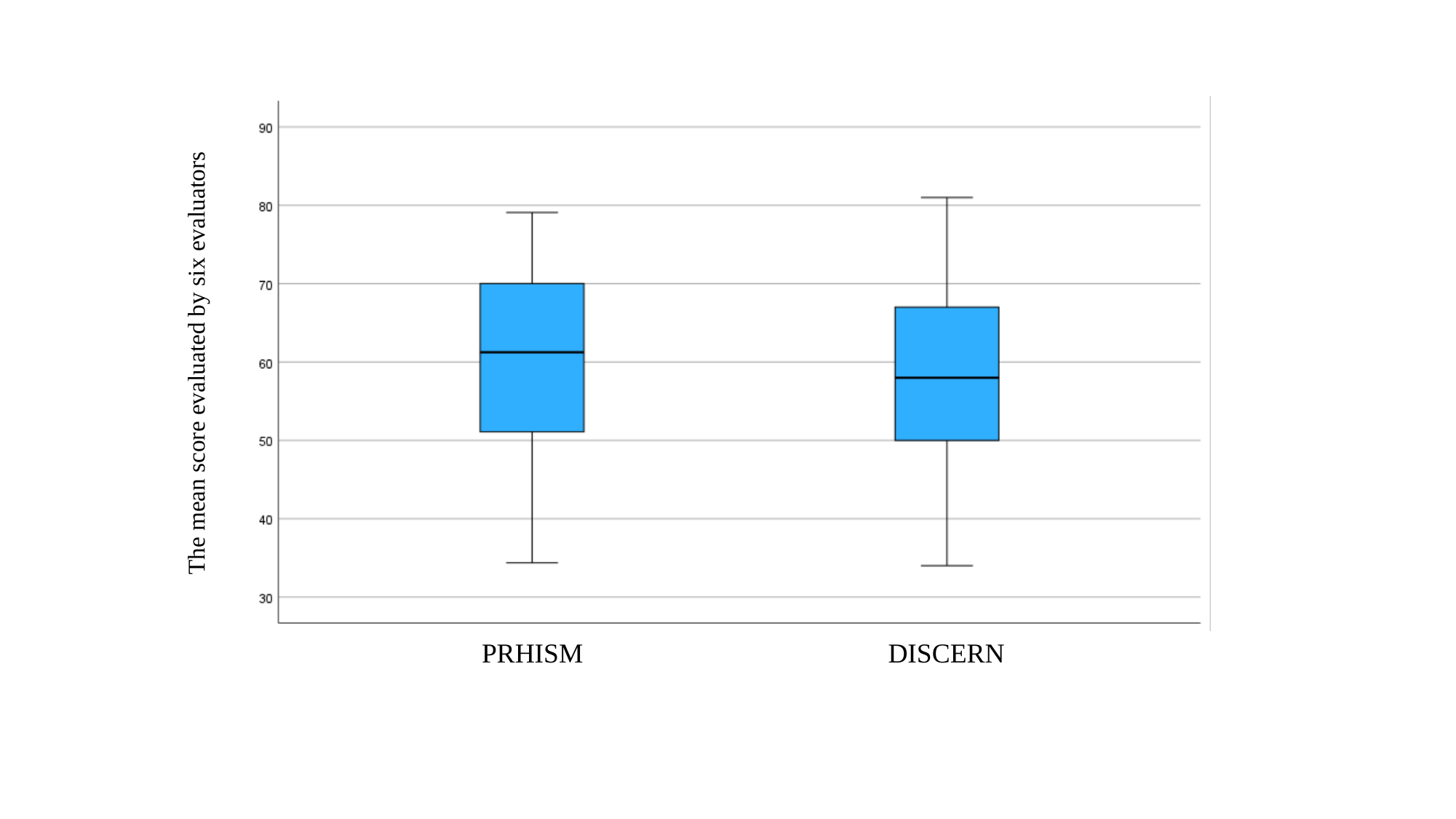

The mean score evaluated by six evaluators
PRHISM
DISCERN

Supplement: Multimedia Appendix 7 [file infodemiology-v5-e66416-s007.pptx]
