# Supplementary material for: Assessing the Reliability and Validity of Principles for Health-Related Information on Social Media (PRHISM) for Evaluating Breast Cancer Treatment Videos on YouTube: Instrument Validation Study
Source: JMIR Infodemiology. 2025 Jun 11;5:e66416. doi: 10.2196/66416 (PMC12175871; doi:10.2196/66416)
Supplement: Multimedia Appendix 4 [file infodemiology-v5-e66416-s004.pptx]

## Slide 1
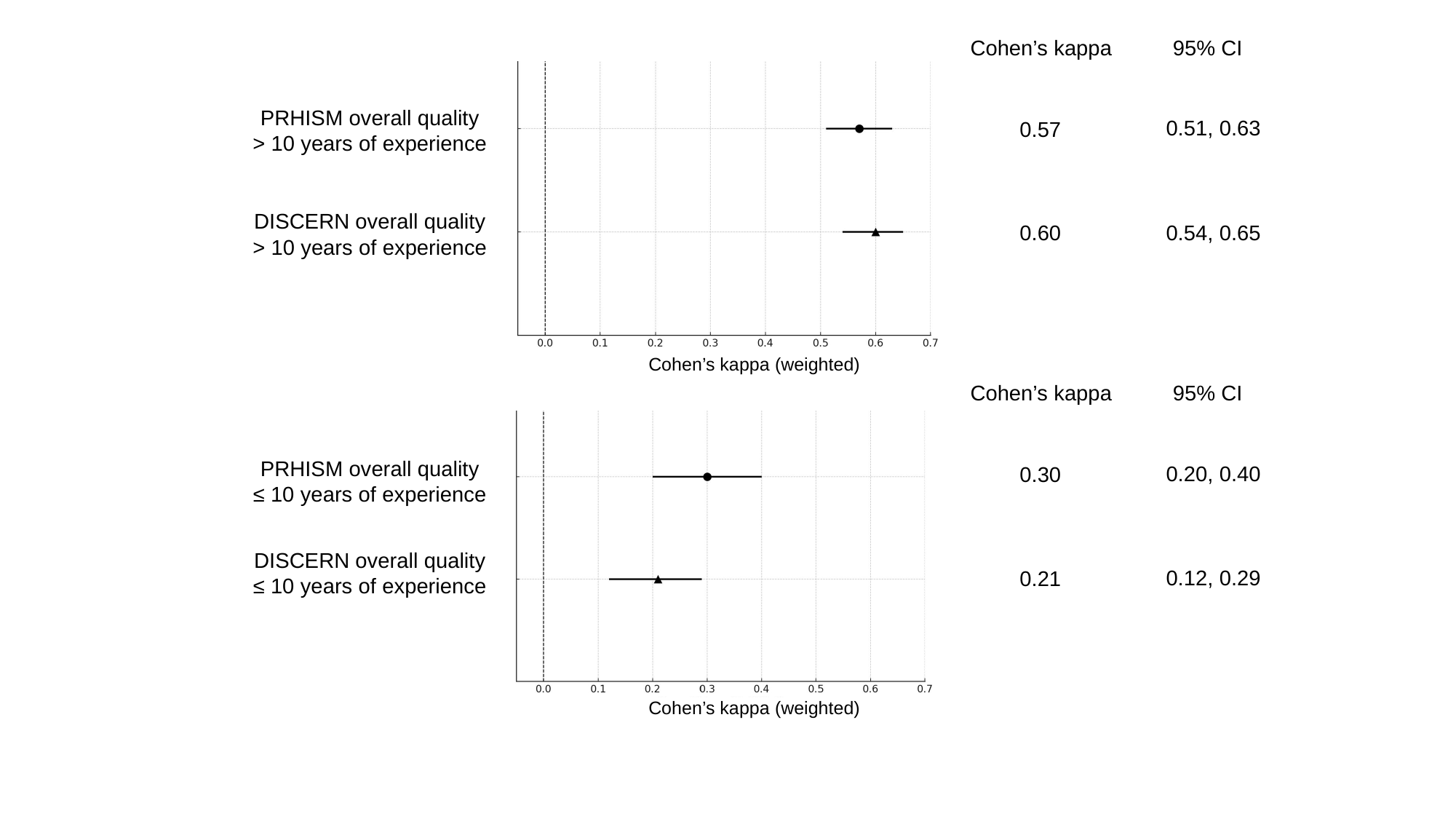

Cohen’s kappa
95% CI
PRHISM overall quality
> 10 years of experience
0.51, 0.63
0.57
DISCERN overall quality
> 10 years of experience
0.54, 0.65
0.60
Cohen’s kappa (weighted)
Cohen’s kappa
95% CI
PRHISM overall quality
≤ 10 years of experience
0.20, 0.40
0.30
DISCERN overall quality
≤ 10 years of experience
0.12, 0.29
0.21
Cohen’s kappa (weighted)
