# Supplementary material for: Assessing the Reliability and Validity of Principles for Health-Related Information on Social Media (PRHISM) for Evaluating Breast Cancer Treatment Videos on YouTube: Instrument Validation Study
Source: JMIR Infodemiology. 2025 Jun 11;5:e66416. doi: 10.2196/66416 (PMC12175871; doi:10.2196/66416)
Supplement: Multimedia Appendix 5 [file infodemiology-v5-e66416-s005.pptx]

## Slide 1
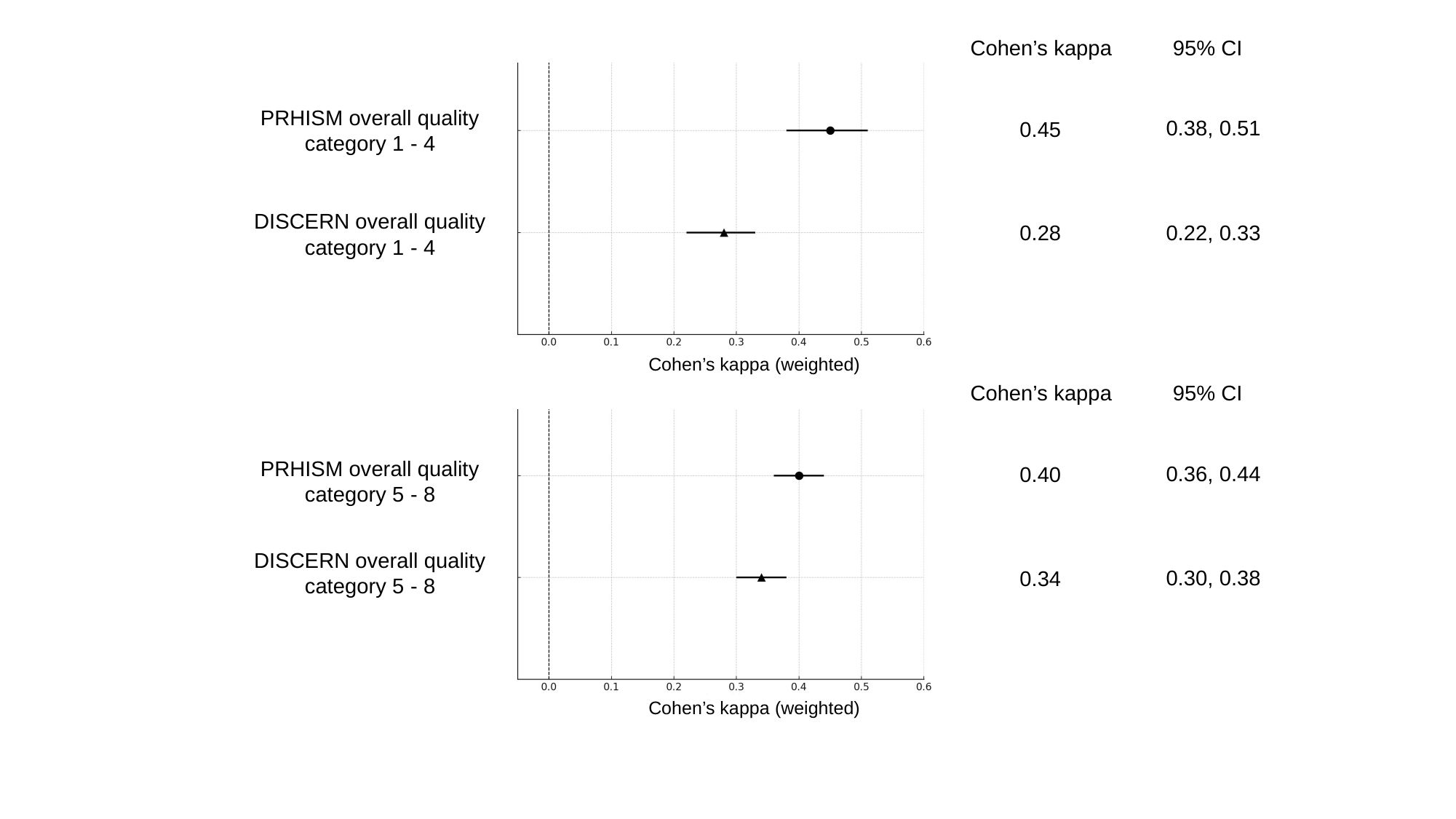

Cohen’s kappa
95% CI
PRHISM overall quality
category 1 - 4
0.38, 0.51
0.45
DISCERN overall quality
category 1 - 4
0.22, 0.33
0.28
Cohen’s kappa (weighted)
Cohen’s kappa
95% CI
PRHISM overall quality
category 5 - 8
0.36, 0.44
0.40
DISCERN overall quality
category 5 - 8
0.30, 0.38
0.34
Cohen’s kappa (weighted)
